# Shape Analysis as an Additional Tool in Roe Deer (*Capreolus capreolus*) Management: A New Approach Based on the Relationship between Mandible Shape and Trophic Resources

**DOI:** 10.3390/ani11061611

**Published:** 2021-05-29

**Authors:** Cesare Pacioni, Francesca Mercati, Andrea Catorci, Andrea Brusaferro, Diederik Strubbe, Paola Scocco

**Affiliations:** 1 Terrestrial Ecology Unit, Department of Biology, Faculty of Sciences, Ghent University, 9000 Ghent, Belgium; diederik.strubbe@ugent.be; 2 Department of Veterinary Medicine, University of Perugia, 06126 Perugia, Italy; 3 School of Biosciences and Veterinary Medicine, University of Camerino, 62032 Camerino, Italy; andrea.catorci@unicam.it (A.C.); paola.scocco@unicam.it (P.S.); 4 FaunLab, Via Ottaviani 46, 62032 Camerino, Italy; andrea.brusaferro@gmail.com

**Keywords:** geometric morphometry, shape analysis, wildlife management, roe deer, mandible, trophic resources

## Abstract

**Simple Summary:**

In recent years, the numerical increase of the roe deer population in Italy has shifted attention to new tools for the management of these populations. The use of biometric data for a correct assessment of the status of ungulate populations is now a standardized and commonly used procedure. In this study, we tried to verify whether, in addition to biometric analyses, morphometrics could be used as a supplementary tool for roe deer management. The study of geometric morphometry has made a major impact by aiding technological and methodological advances. By using Cartesian coordinates of reference points, this method is capable of capturing morphologically distinct shape variables, which can be used as rapid indicators of the status of populations, and thus have the potential to be very useful for wildlife management.

**Abstract:**

The analysis of body shape variability has always been a central element in biology. More recently, geometric morphometry has developed as a new field in shape analysis, with the aim to study body morphological variations and the identification of their causes. In wildlife management, geometric morphometry could be a useful tool to compare the anatomical structures of an organism and quantify its geometric information in order to relate them to environmental factors, thus identifying the causes and effects of the variation and acting management and/or conservation plans. The aim of our study is to evaluate the relationship between roe deer mandible shape and trophic resources available during autumn and winter. We applied a geometric morphometry approach consisting of a Relative Warp analysis of landmark data in 26 roe deer fawn mandibles. Each sample was assigned to an age category and to an environmental category based on the territory carrying capacity. The mandible shape of samples under 8 months of age is likely influenced by the availability of trophic resources. Our findings suggest that the mandible shape is a reliable instrument to assess resource availability. Geometric morphometry could thus represent an additional tool for roe deer management.

## 1. Introduction

The analysis of body shape plays a fundamental role in many biological studies. Morphological characteristics have been key for taxonomic classification and for understanding biological diversity; more recently, geometric morphometry has developed as a new field in shape analysis to study morphological variation and identify its causes, combining plentiful statistical theories [1,2,3,4]. Compared to traditional morphometry, geometric morphometry is a landmark-based method that, instead of linear distance measurements, uses coordinates of points as descriptors of the shape, i.e., ‘landmarks’ [5]. The capture of the geometry of an object using coordinate-based data [3] favours a more comprehensive quantification of biological shape and aids data interpretation through graphical visualization of shape differences. Therefore, it is not surprising that this approach is becoming very common to quantify anatomical shapes in a wide range of scientific applications, such as animal welfare, human evolution, taxonomy, etc. [6,7,8,9,10,11,12,13,14].

Wildlife management however still relies heavily on ‘traditional’ methods and could benefit from adopting more advanced analyses. For this reason, geometric morphometry could be a useful tool to compare the anatomical structures of an organism and quantify its geometric information in order to relate them to environmental factors (e.g., trophic resources), thus identifying the likely causes and effects of the variation and acting management and/or conservation plans.

Such information might be particularly useful in ungulates since these animals respond quickly to environmental changes [15,16]. Inside the Cervidae family, roe deer (*Capreolus capreolus*) is the species with the greatest range in Europe [17]. The diversity of habitats occupied by these animals is the best indication of its success [17]. Roe deer populations are increasing sharply in most of Europe [18], including expansion into lowland areas, due to the decrease of intensive stock-breeding and the abandonment of agricultural lands [19], and their recent expansions have required reliable information on population status and habitat quality, also leading to an increase in size and shape studies [20,21,22,23,24]. In Italy, roe deer represent one of the main species of management interest, not only for selective hunting but also for interactions with human activities. In fact, the increase of roe deer populations often represents the cause of road accidents and significantly raises crop damage [25]. Biometrical data has been recognised to be particularly appropriate for roe deer management [23,26,27]. Their fast body growth [28] makes them a perfect candidate for the study of body parameter and environmental feature relationships; in particular, the mandible is one of the first bones to complete its growth and is therefore sensitive to different environmental factors [15,27]. We used a two-dimensional geometric morphometry approach consisting of a Relative Warp (RW) analysis of landmark data in roe deer fawn mandibles, in a population residing in the Macerata Province (central Italy Apennines). We evaluated the relationship between mandible shape and trophic resources available during autumn and winter—the most critical feeding period for this species [29]—with the aim to contribute to the development of reliable monitoring tools for informing wildlife management strategies.

## 2. Materials and Methods

### 2.1. Study Area

The study was was carried out using mandible samples sourced from the institutions for wildlife management called the ATC-MC2 (Territorial Hunting Zone), responsible for the selective hunting activities in Macerata Province. The ATC-MC2 covers 163,344 ha and has an irregular border naturally delimited to the east by the Adriatic Sea, to the north by the Potenza River, to the west by the Umbria-Marche Apennines and to the south by the Chienti River and the Ete Morto and Salino streams. Each ATC divides its territory into sub-zones called “Management Districts” (MDs) with easily identifiable boundaries that coincide with natural, physical and administrative limits, roads, etc. In turn, each MD is divided into a variable number of “Management Units” (MUs). The ATC-MC2 is divided into 6 MDs and many MUs, based on the density of the roe deer population [29]. From a climatic point of view, the study area belongs to the sub-Mediterranean bioclimatic type, characterized by the alternation of cold winter with summer drought stress [30]. The natural forest vegetation belongs to the *Quercetalia pubescentis-petraeae* order, with the forest mainly dominated by *Quercus punbescens* s.l., *Ostrya carpinifolia* or *Quercus cerris* (*Salix alba* along the rivers). The bushes are most commonly dominated by *Spartium junceum* or different types of Rosaceae [31]. The sub-Mediterranenan climatic features lead to a peak of aboveground phytomass production in spring/early summer, with a long period of vegetative stasis of the ecosystem from November to March.

### 2.2. Data Collection

We used 26 mandibles of roe deer female fawns (aged 0–11 months) sourced from the selective hunting activities in the ATC-MC2. Mandibles were later separated into two groups: 12 samples aged less than 8 months that did not show the third molar, and 14 samples aged 8–11 months that showed the third molar. Each mandible was photographed on the dorsal and ventral sides using a single-lens reflex. Cartesian x, y coordinates of landmarks (marked by specific topographical points) and semi-landmarks (derived relative to the position of other landmarks) were recorded using Geogebra [2], both on the dorsal and ventral sides [5,24]. We considered 19 landmarks on the lateral side and 14 on the dorsal side for mandibles aged 0–8 months, and 20 landmarks on the lateral side and 15 on the dorsal side for mandibles aged 8–11 months. In order to evaluate the relationship between mandible shape and the trophic resources available in the autumn-winter period, we assigned to each sample an environmental category based on the carrying capacity of the MU where the animal was killed, as described in De Felice et al. [29]:

Category 1 = 0—0.02 animals/hectare, with lower food availability;
Category 2 = 0.03—0.1 animals/hectare, with intermediate food availability;
Category 3 = 0.11—0.13 animals/hectare, with higher food availability.

### 2.3. Statistical Analysis

Shape variables were generated by standardising the size, translating, and rotating the landmark configurations using a Generalized Procrustes Analysis [32] with the tpsRelw (thin-plate spline Relative Warps) program [33], which represents a principal component analysis performed on the matrix of shape coordinates after projection in a Euclidean space tangent to the curved shape space. Shape differences were visualised using thin-plate spline deformation grids [34]. The centroid size of each specimen was extracted from the dorsal and the ventral projection of the mandible using tpsRelw. To test for differences among the environmental categories, we carried out ANOVA and discriminant analysis of the Relative Warps (RWs), and the Tukey Test as a post hoc test. The assumptions of normality and homogeneity for the analyses were tested using the Shapiro–Wilk and Levene’s test, respectively. All the statistical analyses were performed using SPSS software.

## 3. Results

TpsRelw on roe deer mandibles generated two consensus configurations (i.e., the average structure obtained from the best landmarks’ overlapping of all samples), one for the dorsal and one for the lateral side. These consensus configurations showed the spatial displacement of each landmark (indicated by vectors) from the mean (average) configuration of landmark coordinates.

**Figure 1:**
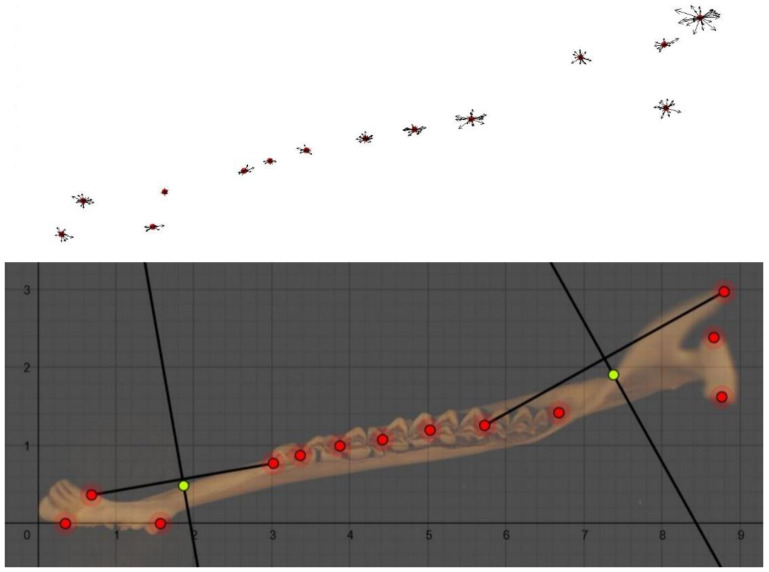
(**On the top**): the consensus configuration for the dorsal side of the mandible (aged 0–8 months). (**On the bottom**): the caption of the dorsal side of the mandible (aged 0–8 months) from Geogebra. Landmarks are shown in red dots, while semi-landmarks are shown in yellow. Semi-landmarks were placed on the anatomical part encountered by the axis perpendicular to the midpoint of the segment connecting two landmarks.

**Figure 2:**
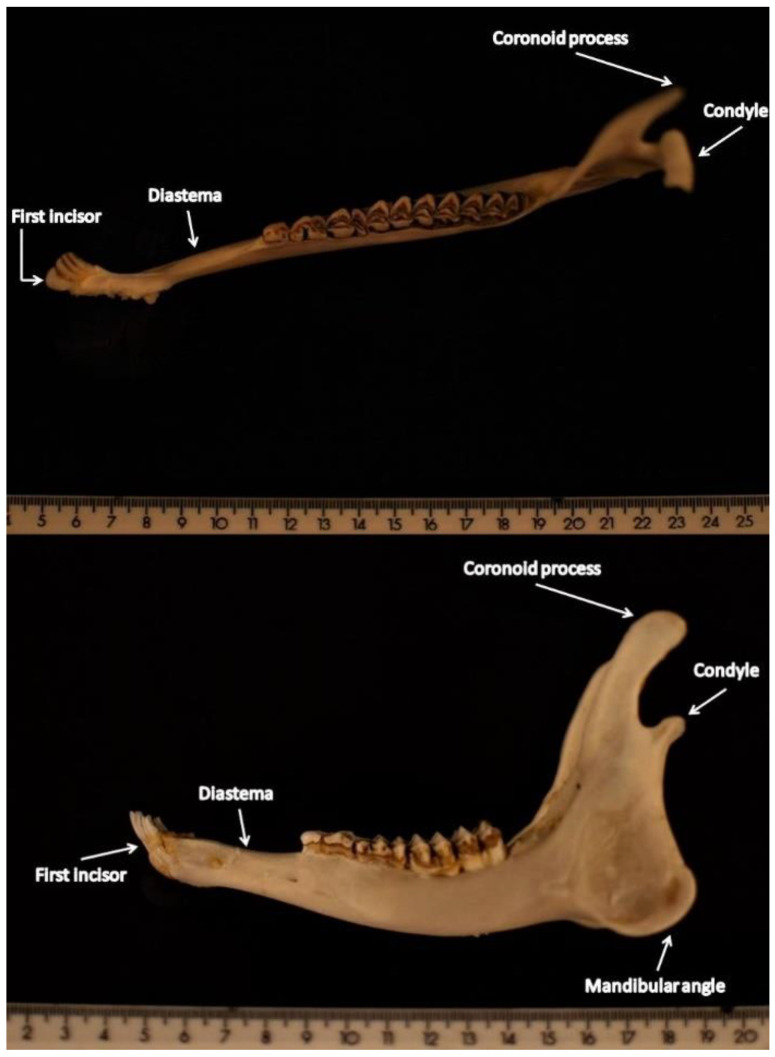
(**On the top**): view of the dorsal side of the mandible. (**On the bottom**): view of the lateral side of the mandible.

Regarding the dorsal side (Figure 1), the landmark with more spatial displacement with respect to the mean configuration corresponds to the posterior limit of the mandible coronoid process (Figure 2).

**Figure 3:**
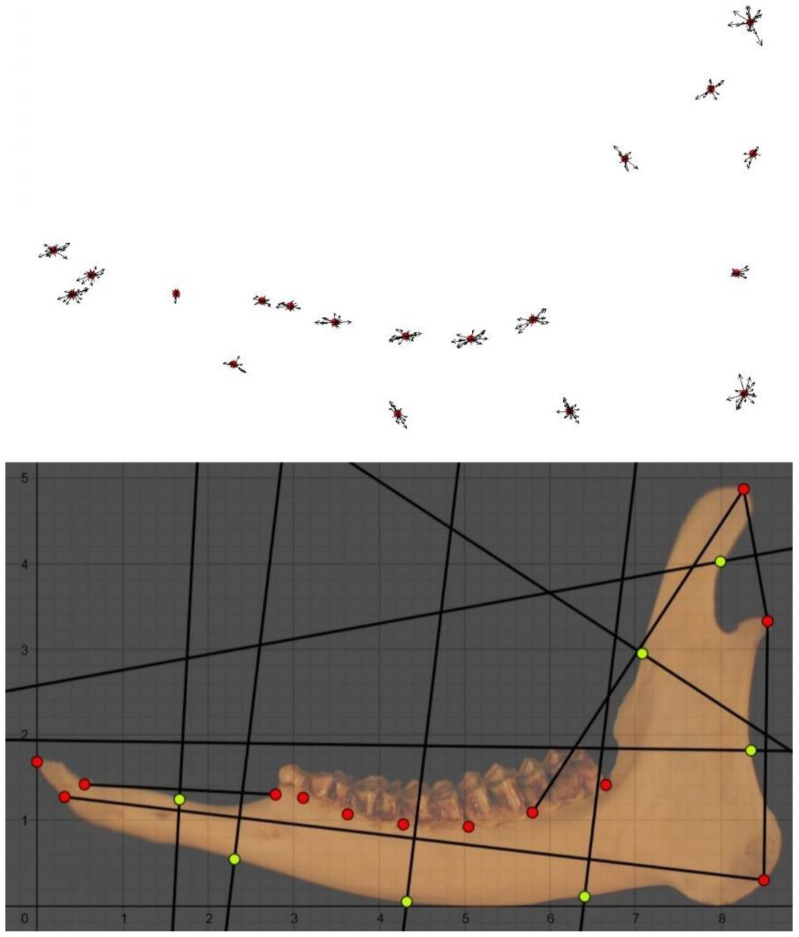
(**On the top**): the consensus configuration for the lateral side of the mandible (aged 0–8 months). (**On the bottom**): the caption of the lateral side of the mandible (aged 0–8 months) from Geogebra. Landmarks are shown in red dots, while semi-landmarks are shown in yellow. Semi-landmarks were placed on the anatomical part encountered by the axis perpendicular to the midpoint of the segment connecting two landmarks.

The consensus configuration for the lateral side (Figure 3) seemed to confirm the same predominant variation among landmarks, in addition to the landmark corresponding to the mandibular angle (Figure 2).

No significant differences (all *p*-values > 0.05) among environmental categories emerged by the statistical analysis of Relative Warp scores from mandibles pertaining to subjects aged 0–11 months, as well as from mandibles pertaining to subjects aged 8–11 months, for either the dorsal or lateral side (Table 1 and Table 2).

Statistical analysis of Relative Warp scores from the dorsal side of mandibles pertaining to subjects aged 0–8 months showed a significant *p* value of 0.025 for the RW1, while for the other components, i.e., RW 2–4, we obtained values of *p* > 0.05, thus demonstrating no significant differences (Table 1). No significant differences were found for the lateral side of mandibles pertaining to subjects aged 0–8 months (Table 2).

The Tukey test of the RW1 (Table 3), although not significant (*p* = 0.053), identifies a variation trend among the three environmental categories regarding the dorsal side.

Relative Warp analysis indicated a dislocation of the environmental categories along RW1, corresponding to the distribution of samples on the basis of the environmental category to which they belong as showed in the graph of Figure 3, in which the 0 point of RW1 corresponds to the mandible dorsal side consensus configuration showing a regular consensus grid. Mandibles belonging to environmental category 3 are mainly located on the left of the 0 point of RW1, while mandibles belonging to environmental category 1 are located on the right of the 0 point of RW1; samples belonging to environmental category 2 are distributed on both sides.

**Figure 4:**
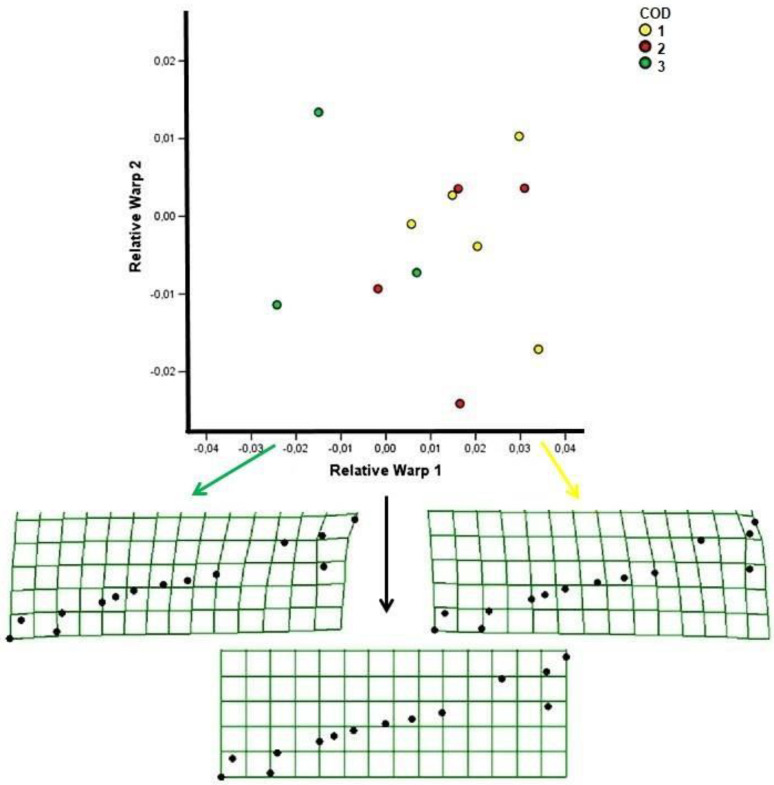
RWs (axis 1 and 2) of mandibles pertaining to subjects aged 0–8 months. Deformation grids evidence the sample shape modification along RW1 with respect to the consensus grid linked to the environmental category of the samples. COD 1, 2, 3 indicates the environmental category.

The deformation grids shown on the left and right in Figure 4 evidenced the sample shape modification along RW1 with respect to the consensus grid between samples of environmental category 3 and 1 respectively; modifications mainly concern the dorsal margin of the mandible ramus corresponding to the coronoid process landmark.

The discriminant analysis did not show significant values.

## 4. Discussion

Assessing the role of the environment in body structure development is fundamental to inform management decisions such as selective hunting. In ungulates, selected bones are regularly used as indicators of environmental changes [35,36] and, among them, the mandible is often a main element [37,38,39,40]. Researchers frequently use mandible as an ecological indicator [41], especially in roe deer, because it is one of the first bones to ossify and to reach its final size within the early years of life [42,43]. Thus, it can be expected that the development of the mandible of fawns is particularly sensitive to the nutritional conditions experienced during the early stages of growth. In the present work, only mandibles from female roe deer were used; however, this fact does not compromise the results as it has been proven that there are no biometrical differences between sexes in roe deer fawns [29].

The significant variation on the landmark of the coronoid process in the bone samples pertaining to roe deer assigned to different environmental categories indicated that the mandible shape of samples aged 0–8 months is likely influenced by the availability of trophic resources. Roe deer are ‘concentrated selectors’, as their diet is based on the choice of plant species (or their parts) with high cellular juice and limited fibre contents, such as sprouts, buds, young leaves, mainly dicots grasses, fruits and seeds [44,45]. The autumn-winter period represents the most critical period for roe deer feeding for two reasons: the plant vegetative stasis (when the availability of selected feed is scarce) and the snow covering [46]; therefore, these two factors represent the main elements for the assessment of habitat quality for roe deer.

In our study, environmental category 3 represents the MU territories with a high forest covering likely to provide buds and seeds during the critical period for roe deer, while environmental category 1 represents the MU territories with a high presence of pasture or cultivated fields, which during the winter are unsuitable for roe deer feed [29].

As the deformation grids showed modifications mainly concerning the coronoid process landmark, we can say that samples belonging to environmental category 3, with its higher feed availability [29], showed significantly more open mandibular angles compared to the samples belonging to environmental category 1, which offers the lower feed availability. Hence, food availability during the autumn-winter period seems to represent a critical factor for the development and morphology of this bone structure in the earliest life stage of roe deer. The effect of feed availability on the development of roe deer mandibles is further supported by a previous study on size analysis of adult roe deer, where the teeth row length was found to be significantly longer for samples pertaining to a better environmental category [29].

Unfortunately, the coronoid process, being a very protruding part, is easily damaged or lost during sample preparation, so that analysis could not always take into account this part in the study of mandible shape or size [39]. Despite the difficulty of having intact mandibular samples, the coronoid process must be taken into consideration, according to us, to monitor and evaluate the relationship between trophic resources and roe deer mandible shapes, and above all for the roe deer fawn mandible samples. In fact, generally, there were few fawns among the selected shooting subjects; in addition, those pertaining to environmental category 3 are the least represented, due to the fact that they live in a more optimal and protective home range [29,46].

Geometric morphometry could therefore be a good approach to complement the study and management of roe deer populations. It allows the study of the overall morphological variation of a shape and is an excellent tool for quantifying and representing the variation in different structures. Furthermore, the use of the software is very useful in simplifying the morphometric analysis and to better visualise the results. For example, the expansion or contraction of the deformation grid created by the software shows which part of the organism is changing compared to the others, and the visualisation of the change in shape is potentially informative from a biological point of view.

## 5. Conclusions

A recent study [29] showed a relationship between food availability during the autumn-winter period and some roe deer body parameters. Specifically, the length of the hook seemed to be affected by trophic resources in both fawns and adult roe deer. This evidence allows us to consider the length of the hook as a potential predictive parameter of habitat quality. In the same way, our shape analysis could help to identify other bone structures whose variation could be predictive of environmental changes, adding useful information to those provided by size analysis.

The long-term monitoring of geometric morphometry data may provide information on how these structures change, and which part of every single structure examined is more predisposed to environmental factors. This approach clearly deserves more investigation in order to apply it as a tool to define body marker parameters related to environmental carrying capacity, with the aim to plan the roe deer population management based on the shape analysis of the samples collected from the selective shooting of the previous years.

## Figures and Tables

**Table 1:** ANOVA One-way results from dorsal side data.

Dorsal Side		0–11 Months Old (N = 26)					8–11 Months Old (N = 14)					0–8 Months Old (N = 12)				
		SS	df	MS	F	*p*	SS	df	MS	F	*p*	SS	df	MS	F	*p*
RW1	BG	0.001	2	0.000	0.588	0.563	0.001	2	0.000	0.530	0.603	0.002	2	0.001	5.737	0.025
	WG	0.012	23	0.001			0.005	11	0.000			0.002	9	0.000		
	Total	0.012	25				0.006	13				0.004	11			
RW2	BG	0.001	2	0.000	1.720	0.201	0.001	2	0.001	3.627	0.062	0.000	2	0.000	0.216	0.810
	WG	0.004	23	0.000			0.002	11	0.000			0.001	9	0.000		
	Total	0.004	25				0.003	13				0.001	11			
RW3	BG	0.000	2	0.000	0.336	0.718	0.000	2	0.000	0.597	0.567	0.000	2	0.000	0.460	0.646
	WG	0.003	23	0.000			0.001	11	0.000			0.002	9	0.000		
	Total	0.004	25				0.001	13				0.002	11			
RW4	BG	0.000	2	0.000	0.583	0.566	0.000	2	0.000	0.469	0.637	0.000	2	0.000	0.207	0.816
	WG	0.001	23	0.000			0.001	11	0.000			0.001	9	0.000		
	Total	0.001	25				0.001	13				0.001	11			

N = number of samples; SS = sum of squares; df = degrees of freedom; MS = mean square; RW = relative warp; BG = between groups; WG = within groups.

**Table 2:** ANOVA One-way results from lateral side data.

Lateral Side		0–11 Months Old (N = 26)					8–11 Months Old (N = 14)					0–8 Months Old (N = 12)				
		SS	df	MS	F	Sig.	SS	df	MS	F	Sig.	SS	df	MS	F	Sig.
RW1	BG	0.002	2	0.001	0.895	0.121	0.002	2	0.001	1.506	0.264	0.000	2	0.000	691	0.526
	WG	0.011	23	0.000			0.008	11	0.001			0.001	9	0.000		
	Total	0.013	25				0.011	13				0.002	11			
RW2	BG	0.000	2	0.000	2.321	0.611	0.000	2	0.000	0.853	0.452	0.000	2	0.000	0.120	0.889
	WG	0.005	23	0.000			0.002	11	0.000			0.003	9	0.000		
	Total	0.005	25				0.003	13				0.003	11			
RW3	BG	0.000	2	0.000	0.503	0.434	0.000	2	0.000	1.112	0.363	0.000	2	0.000	0.144	0.867
	WG	0.003	23	0.000			0.001	11	0.000			0.001	9	0.000		
	Total	0.003	25				0.001	13				0.001	11			
RW4	BG	0.000	2	0.000	0.910	0.417	0.000	2	0.000	0.241	0.790	0.000	2	0.000	0.639	0.550
	WG	0.002	23	0.000			0.001	11	0.000			0.001	9	0.000		
	Total	0.002	25				0.001	13				0.001	11			

N = number of samples; SS = sum of squares; df = degrees of freedom; MS = mean square; RW = relative warp; BG = between groups; WG = within groups.

**Table 3:** Tukey HSD results from dorsal side data from samples pertaining to animals aged 0–8 months.

COD	N	Subset for Alpha = 0.05	
		1	2
3	3	−0.0108	
2	4	0.0154	0.0154
1	5		0.0209
*p*	0.053	0.836	

COD 1, 2, 3 indicates the environmental category; N = number of samples.

## Data Availability

The data presented in this study are available on request from the corresponding author.
